# Helping Mothers Survive: Program Evaluation and Early Outcomes of Maternal Care Training in the Dominican Republic

**DOI:** 10.3389/fpubh.2021.660908

**Published:** 2021-06-16

**Authors:** Meghan Smith, Alexandra Leader, Wanny Roa, Ericka Jaramillo, Davina Lazala, Jose Flores, Claudia Cadet, Turaj Vazifedan, Suzanne Bentley, Lloyd Jensen

**Affiliations:** ^1^Graduate Program in Public Health, Icahn School of Medicine at Mount Sinai, New York, NY, United States; ^2^Department of Pediatrics, Eastern Virginia Medical School, Norfolk, VA, United States; ^3^Region II, Ministry of Health, Santiago, Dominican Republic; ^4^Family Medicine, Natividad Medical Center, Salinas, CA, United States; ^5^Department of Family Medicine, University of Washington, Seattle, WA, United States; ^6^Department of Neonatology, WakeMed Health and Hospitals, Raleigh, NC, United States; ^7^Department of Pediatrics, Children's Hospital of the King's Daughters, Norfolk, VA, United States; ^8^Department of Emergency Medicine, Elmhurst Hospital Center, Elmhurst, NY, United States; ^9^Department of Pediatrics, University of Nevada Las Vegas, Las Vegas, NV, United States

**Keywords:** maternal mortality, COVID-19, simulation-based training, helping mothers survive, maternal health, pre-eclampsia & eclampsia, postpartum hemorrhage

## Abstract

**Introduction:** In 2017, approximately 295,000 women died during and immediately following pregnancy and childbirth worldwide, with 94% of these deaths occurring in low-resource settings. The Dominican Republic (DR) exhibits one of the highest maternal mortality ratios in the region of Latin America and the Caribbean despite the fact that 99% of registered births in the country are reportedly attended by a skilled birth attendant. This paradox implies that programs to support healthcare worker knowledge and skills improvement are vital to improving maternal health outcomes in the DR. Helping Mothers Survive (HMS) is a provider training program developed by Jhpiego and global partners. The goal of HMS is to combat maternal mortality by contributing to quality improvement efforts that reinforce maternal health skills of local healthcare workers.

**Methods:** An international, multisectoral group of stakeholders collaborated in the implementation of two HMS curricula, Bleeding After Birth (BAB) and pre-eclampsia & eclampsia (PE&E). Demographic information as well as pre- and post-training knowledge scores were recorded for each participant. Knowledge score improvement was assessed in order to support effectiveness of the program on knowledge acquisition of healthcare workers.

**Results:** Three hundred and twenty healthcare workers participated in the HMS training workshops between October 2016–August 2020. Of the 320 participants, 132 were trained as master trainers. The majority of participants identified as attending physicians, followed by residents/interns, nurses, students, and “other.” A significant improvement in knowledge scores was observed for both the BAB and PE&E curricula, with a 21.24 and 30.25% change in average score (pre- to post-test), respectively. In response to COVID-19 pandemic restrictions, flexibility of the local team led to a PE&E virtual training pilot workshop in August 2020.

**Discussion/Conclusions:** Simulation-based training improved the knowledge levels of healthcare workers for both HMS curricula. These results suggest that simulation-based workshops have an impact on knowledge acquisition and skills of healthcare workers immediately following training. For the PE&E curriculum, no significant difference in knowledge acquisition was observed between in-person and virtual training sessions. The ongoing pandemic poses challenges to program implementation; however, these preliminary results provide evidence that conducting virtual workshops may be a viable alternative to in-person training.

## Introduction

Global maternal mortality remains concerningly high despite ongoing public health efforts. According to the most recent data, in 2017 approximately 295,000 women died during and immediately following pregnancy and childbirth worldwide, with 94% of these deaths occurring in low and middle-income countries (1). Principal causes of global maternal death are postpartum hemorrhage (PPH), pre-eclampsia/eclampsia, infections after childbirth, complications during birth, and unsafe abortion, all of which are preventable with proper access to trained healthcare workers (1). Past maternal mortality prevention strategies have failed to achieve the maternal health goals outlined by the United Nations Millennium Development Goals of 2000, such as the reduction of the global maternal mortality ratio by three quarters by the year 2015 (2). In efforts to address these shortcomings and create accountability in the area of maternal health, target 3.1 of the United Nations Sustainable Development Goals was created with the goal of decreasing the worldwide maternal mortality ratio to <70 deaths per 100,000 live births by 2030 (3).

The average maternal mortality ratio for Latin America and the Caribbean was 74 deaths per 100,000 live births in 2017 (4). Although the region of Latin America and the Caribbean appears to have favorable statistics on maternal mortality compared to other regions, specifically Sub-Saharan Africa, the maternal mortality ratio in the Dominican Republic (DR) is particularly high, with 95 deaths per 100,000 live births in 2017 (5). According to more recent reports from the Dominican Ministry of Health's National Epidemiological Vigilance System, the maternal mortality ratio continues to increase, with the maternal mortality ratio reported as 113 deaths per 100,000 live births in 2018 (6). In the DR specifically, the two leading causes of maternal mortality as of 2017 were hemorrhage (23% of deaths) and pre-eclampsia & eclampsia (PE&E) (22.1% of deaths) (7). This high maternal mortality ratio persists despite the fact that a surprising 99% of registered births in the DR (as of 2015) were reportedly attended by a skilled healthcare worker (8). This paradox implies that a deficit in healthcare worker competencies and/or standardized treatment protocols specific to these common causes of maternal mortality may contribute to high maternal mortality rates in the DR.

In an effort to address competency deficits and access to standardized management protocols among frontline healthcare workers attending births, sustainable maternal care training programs must be implemented. Helping Mothers Survive (HMS) is a simulation-based training curriculum developed by Jhpiego in collaboration with global partners, with the goal of combating maternal mortality through the improvement of maternal care skills among local healthcare workers. Helping Mothers Survive was modeled after the Helping Babies Breathe (HBB) training program, which has been shown to improve birth attendants' neonatal resuscitation knowledge and skill levels as well as decrease infant mortality in various low-resource settings (9–11). Helping Mothers Survive is composed of four training modules focusing on PPH, pre-eclampsia/eclampsia, essential care for labor and birth, and threatened preterm birth care (12). These training packages utilize a train-the-trainer (T3) approach, training a cohort of master trainers who then train additional healthcare workers in their respective healthcare settings. The implementation of HMS and similar simulation-based training programs has been associated with successful knowledge and skill acquisition, as well as improved clinical outcomes, among healthcare workers in multiple global settings (13–16). A study evaluating HMS Bleeding After Birth (BAB)-trained healthcare workers in 20 districts in Tanzania in 2015–2016 found that a one-day competency-based training workshop followed by weekly drills with participants was associated with a statistically significant reduction in rates of severe maternal morbidity related to PPH (13). Similarly, a different study in Northern Tanzania in 2011–2013 observed a significantly reduced incidence of PPH as well as an increased use of skills related to birth and PPH management by healthcare workers who received HMS BAB training (14). In terms of the specific impact of HMS training on healthcare workers' knowledge acquisition, a HMS BAB training program implemented in Northern Tanzania in 2012 found that mean knowledge scores, simulation scores, and confidence levels of the health workers improved after participating in the HMS training modules (15). Moreover, promising results were seen in an evidence-based practice training program in Bangladesh in 2016–2017, which compared baseline health outcomes data to post-training health outcomes data. The program evaluation found significant improvements in the management and diagnosis of pre-eclampsia/eclampsia among training participants (16). These findings support the idea that implementation of simulation-based training programs can significantly impact maternal-infant healthcare worker knowledge, skills, and practices in low-resource settings.

With the intention of broadening and strengthening the knowledge, skills, and practices of frontline healthcare workers in the DR, the HMS curricula was utilized to begin master training in the cities of Santiago and Barahona in 2016. The HBB program was initially implemented in Santiago in 2015 through collaborative efforts of the Dominican Ministry of Health, Latter-day Saint Charities, the Manos de Ramon Foundation, and the Pan American Health Organization (PAHO). These previously established partnerships and existing infrastructure facilitated subsequent implementation of the HMS curricula in this region. Two of the four HMS modules were identified by the Ministry of Health and local healthcare professionals as the most relevant to the regional challenges in maternal health: BAB and PE&E. Master training for BAB began in Santiago, DR in October 2016, while follow-up training began in the same region 2 months later in December 2016. Master training for PE&E began in Barahona in October 2018. Local hospital administrators selected the initial cohort of master trainers by identifying local champions who would prioritize engagement in training sessions and subsequent implementation of the workshops.

The following HMS program evaluation will assess programmatic elements such as site of training implementation, entities involved, sources of funding, and how the program was sustained. Further, the evaluation will explore summary statistics of early outcomes regarding number of participants, specialties represented, number of training sessions held, and knowledge acquisition after training. This evaluation aims to examine the impact of the training on participant knowledge levels and identify areas of success and targets for improvement of the HMS curricula in this setting. Due to the dearth of data regarding maternal care training implementation and outcomes in this region, the results of this program evaluation will allow for quality improvement of programming in the DR and may act as a framework for implementing future maternal health training programs in the DR and, more generally, across Latin America and the Caribbean.

## Methods and Materials

This evaluation will assess the implementation process and early outcomes of two modules of the HMS curricula: BAB and PE&E. The evaluation will focus on healthcare worker knowledge and skill development in the regions studied in the DR.

### Site Description and Selection

The maternal care training program focused on implementation in the Enriquillo region and the Cibao regions of the DR. These two regions were selected for implementation due to the successful rollout of the HBB initiative in the same areas beginning in 2015. Previously existing partnerships with local hospitals in these regions facilitated the addition of maternal resuscitation training and use of the HMS curricula to ongoing neonatal resuscitation training.

### Partnerships

A multisectoral group of stakeholders collaborated in the implementation of the HMS BAB and PE&E training curricula. The Dominican Ministry of Health, Latter-day Saint Charities, the Manos de Ramon Foundation NGOs, the PAHO, and local hospital leadership all contributed to the rollout of this initiative. In February 2018, one of the original HBB master trainers, a local Dominican family physician, became a program leader and assumed responsibility for directing ongoing training implementation, providing regional support to master trainers, and conducting follow-up interviews with program participants.

### Training and Equipment

A train-the-trainer (T3) approach was used for the implementation of the HMS BAB and PE&E curricula. The HMS BAB curriculum aims to train providers to master competencies surrounding the prevention, detection, and management of PPH. The materials associated with the training package include: (1) Action Plans, (2) The Flip Chart, (3) A Provider's Guide, and (4) The Mama Natalie Birthing Simulator (17). Training begins with a didactic learning session, followed by the opportunity for participants to take part in simulated scenarios with their colleagues as a means to practice incorporating the newly improved or acquired skills. These simulations were facilitated by the use of Mama Natalie, a low-tech birthing simulator that is able to model a variety of birthing complications, including PPH (18). The use of Mama Natalie is vital to the development of skills that can be translated into everyday clinical practice. Upon completion of the training, with iterative practice until successful acquisition of skills was observed by course facilitators, a representative course participant from each training site was provided with training and simulation materials and laminated medical treatment algorithms to display in clinical areas of the hospital. In December 2016, due to generous contributions from Latter-day Saint Charities, these materials were distributed to the local participating hospitals with the intention that they would not only facilitate sustainability of training in each master trainer's own clinical setting, but also be integrated widely into everyday clinical practice. Distribution of clinical and training materials was followed by a pilot HMS training with an OB/GYN residency training program in Santiago, which served as an informative foundation for subsequent widespread rollout of HMS training.

The HMS PE&E training was implemented in a similar fashion, with a goal of participant mastery in the prevention, detection, and management of PE&E. The first element of course content targets frontline healthcare workers who may be the first to see women experiencing signs and symptoms of PE&E and includes standardized protocols for initial assessment, diagnosis, and initiation of treatment (19). The second element of the course content is centered on advanced, ongoing care and treatment for women with severe PE&E. Other materials used to support the PE&E curriculum are the trainer's Flip Chart, and a Provider's Guide. Similar to HMS BAB, upon completion of training and demonstration of skill acquisition to the course facilitators, each master trainer received training materials and clinical supplies for integration into professional practice in each clinical setting.

### Healthcare Workers and Maternal Care in the DR

In the DR public healthcare system, births are most commonly attended by general practitioners and accompanying nurses, followed by resident physicians if in a training hospital. In the private healthcare system, gynecologists, pediatricians, and accompanying nurses are available to attend births, perform cesarean sections, and assist with other serious birth complications. The attendance of nurses during childbirth as opposed to midwives varies considerably from other regions of the world with high maternal mortality ratios. Due to the predominant presence of nurses during childbirth in the DR, the HMS program aimed to train and recruit a variety of healthcare workers.

### Data Collection

Data were collected at each site at two different timepoints—during each training workshop and during a subsequent follow-up period. The follow-up periods for the two courses varied between 2 and 6 months due to feasibility of logistics for the local program staff. Questionnaires administered during the master training sessions included basic demographic information (clinical role, length of career in said role, and health site which the participant was representing). Pre- and post-test knowledge assessments were administered prior to and immediately following each training session. During the follow-up period of 2–6 months post-training, formal surveys were also administered to collect information regarding the total number of participants trained in subsequent workshops conducted by master trainers at each hospital training site. Data was collected from each training site by local team members and recorded on Microsoft Excel spreadsheets.

Standardized knowledge assessments were administered at both timepoints at each site. Similar to the HMS training materials outlined previously, the standardized knowledge assessments administered to participants were developed and validated by Jhpiego. For the HMS BAB curriculum, the knowledge pre-/post-test consist of 15 standardized multiple-choice questions, while the knowledge pre-/post-test for the HMS PE&E curriculum has 20 multiple choice questions. Questions were related to course content of the training courses, including diagnosis, prevention strategies, and treatment methods of BAB and PE&E. These data were utilized to compare pre- and post-training knowledge scores per participant and evaluate knowledge acquisition during the course and knowledge retention several months after course completion.

### Adaptations Due to the COVID-19 Pandemic

This HMS program evaluation was conducted remotely in the DR and the United States due to travel restrictions and health concerns surrounding the COVID-19 pandemic. All available de-identified data was shared electronically among Dominican and US collaborators in order to perform analysis. Due to pandemic protocols, there were no in-person training workshops conducted from March 2020 to August 2020, at which point virtual training was piloted.

In August 2020, the first virtual training workshop was conducted in the setting of the emerging COVID-19 pandemic. A group of 13 resident physicians from Hospital CEAS Juan XXIII were trained with the PE&E curriculum via the Google Hangouts video platform. The pre-test was administered the day before the scheduled training session, and each participant was given the course materials to study in advance of the virtual session. The virtual training session itself consisted of a 2-h didactic review of the course material, as well as video simulations. The knowledge post-test was administered after the training via Google Forms with the results recorded on an Excel spreadsheet. The preliminary data from this pilot training workshop are included in the present analysis.

### Statistical Analysis

De-identified data were transferred from Microsoft Excel spreadsheets into the REDCap database in preparation for statistical analysis. Statistical tests were performed using SPSS 26 (Chicago, IL). Descriptive statistics were calculated for participant demographics. Knowledge score data was analyzed using score percentages in order to account for the difference in total questions between the BAB and PE&E knowledge assessments (15 total questions and 20 total questions, respectively). Pre- and post-training knowledge scores were compared using a paired t-test and Wilcoxon signed-rank test. Effect of pre-trainings score, courses (BAB or PE&E), profession, and training sites on score improvements were analyzed using a Generalized Linear Model (GLM). Statistical tests were two-sided and results yielding *p* < 0.05 were considered statistically significant. Summary data regarding the number of subsequent participants trained by master trainers from the follow-up surveys were manually calculated using Excel spreadsheets.

### Ethical Considerations

The HMS training program was developed based on the latest evidence in clinical care with collaboration and input from the International Confederation of Midwives (ICM), the International Federation of Gynecology and Obstetrics (FIGO), the International Council of Nurses (ICN), the American Academy of Pediatrics (AAP), and the United Nations Population Fund (UNFPA) (18). The implementation of HMS training programs in the DR was approved and coordinated by the Dominican Ministry of Health and hospital leadership at all training sites. The study protocol for this program evaluation was approved by the Institutional Review Board of Eastern Virginia Medical School.

## Results

**Figure 1:**
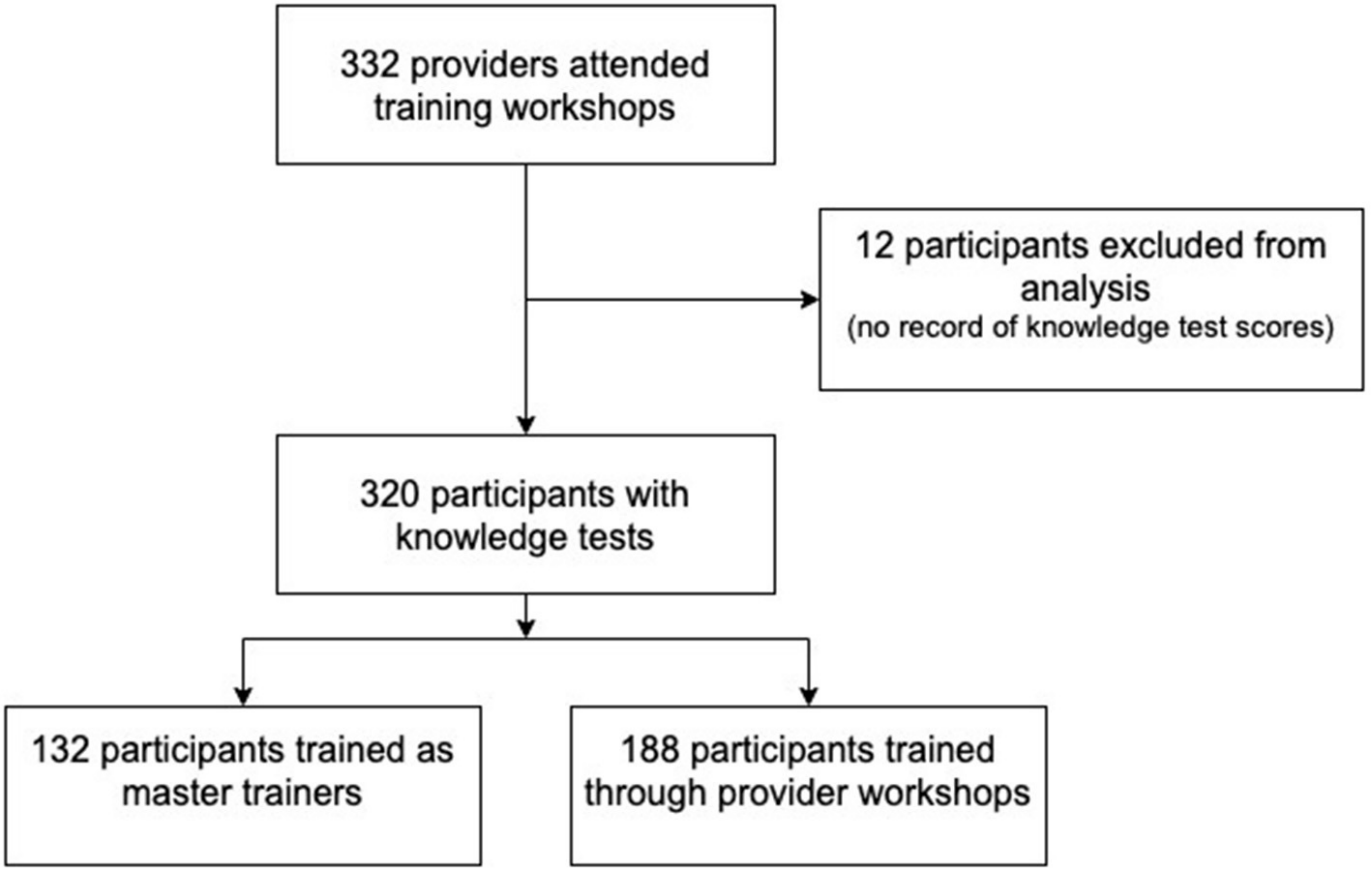
Flow chart of total participants into master trainers vs. general participants.

Three hundred and thirty-two total healthcare workers attended HMS training workshops between October 2016 and August 2020. Of the 332 in attendance, 12 participants were not included in the analysis as they either assisted in the facilitation of the training or did not complete a knowledge pre- and post-test. Out of the 320 participants included in the analysis, 132 (41.3%) were trained as master trainers at one of three master training sites: Santiago, Barahona, and Santo Domingo (See Figure 1). These three masters training sites were among the ten total sites included in the evaluation. One of the ten sites was associated with a virtual training workshop conducted in August 2020. Although the hospital associated with the virtual training was a previously existing training site, the virtual workshop was evaluated separately due to the different experiences and educational content associated with in-person and virtual training, including the limitations in performing simulated skill-based scenarios on a virtual platform.

In regard to the topic of training, the majority of participants attended the PE&E workshops, with 224 (70%) total participants for the PE&E curriculum and 96 (30%) total participants for the BAB curriculum. Four participants (1.25%) participated in both the BAB and PE&E curriculum. Out of the 10 training sites, the site with the largest number of participants was CEAS Juan XXIII with 64 participants (20%), immediately followed by the Santiago master training site with 63 participants (19.7%) (See Table 1).

**Table 1:** Breakdown of number of participants per training site.

**Training site**	**Number of participants**	**Percentage of total participants (%)**
CEAS Juan 23	64	20.0
Santiago Master Training Site	63	19.7
Barahona Master Training Site	50	15.6
Hospital Bella Vista	28	8.8
Instituto Politécnico Rafaela Perez	25	7.8
Hospital San José de las Matas	24	7.5
Hospital Padre Fantino Monte Cristi	20	6.3
Santo Domingo Master Training Site	19	5.9
Hospital Cien Fuego	14	4.4
VIRTUAL CEAS Juan 23	13	4.1

The majority of participants identified themselves as attending physicians (*n* = 137, 42.8%), followed by nurses (*n* = 56, 17.5%), residents/interns (*n* = 49, 15.3%), medical assistants (*n* = 43, 13.4%), and nursing and medical students (*n* = 28, 8.8%). Other professions were represented, such as midwives, teachers, and “other” which included Ministry of Health representatives, auditors, and community health promoters. Table 2 provides a detailed list of all professions represented at the workshops.

**Table 2:** Categories of professions and their respective mean overall % change in knowledge score.

**Group**	**Professions included (*n*)**	**Mean overall % change from pre-test to post-test (%)**
Attending physicians	Internal medicine (27) Family medicine (6) General medicine (6) OB-GYN (3) Unspecified specialty (95)	25.24
Nurses	Nurses (56)	26.95
Residents/Interns	Residents (21) Interns (28)	29.58
Medical assistants	Medical assistants (43)	33.78
Nursing and medical students	Nursing student (25) Medical student (3)	27.41
Other	Other (6) Midwife (1)	29.00

Knowledge improvement was observed for all participants immediately following training. An overall significant change in average score was observed from pre-test to post-test (from 51.7 to 79.3%), resulting in a 27.6% change (*p* < 0.001). When looking at the scores per HMS curriculum, both yielded a statistically significant improvement. The percent change between pre- and post-test scores associated with the BAB curriculum was 21.24%, while the pre- and post-test scores associated with the PE&E curriculum showed a larger improvement of a 30.25% change. Investigation of this difference in score improvement showed that the mean pre-test or baseline scores for BAB were significantly higher (*p* < 0.001) than the mean pre-test scores for PE&E, resulting in less opportunity for improvement for the BAB curriculum. Analysis of the impact of the course type (BAB or PE&E) on score improvement confirmed a significant difference between the courses (*p* = 0.001).

Among the 10 training sites, the site with the greatest score improvement was Hospital Padre Fantino Monte Cristi, with a mean percent change of 39.23% (95% CI 31.64, 46.82). Conversely, the cohort that showed the least improvement was the virtual CEAS Juan XXIII site, with a mean improvement of only 22.69% (95% CI 15.98, 29.41). Although the virtual site was least improved, observed mean PE&E pre-test scores were significantly higher (*p* < 0.001, 95% CI 0.232, 0.568) than all other PE&E training cohorts, likely influencing the minimal change in score observed. A similar trend was observed between the three master training sites and the remaining six provider sites. Average knowledge improvement associated with provider training sites was greater than the average knowledge improvement associated with the master training sites, while the average pre-test scores were greater for master training sites than provider training sites.

While 13 total professions were included in the analysis, the most prevalent professions among participants were physicians, nurses, and interns and residents (Table 2). The greatest improvement in average knowledge score among these groups was associated with family medicine physicians (“*medicos familiares*,” *n* = 6), demonstrating a 36% mean difference between pre- and post-test scores. Upon grouping the 13 professions into six more general categories (physicians, interns & residents, medical assistants, nurses, nursing & medical students, other), knowledge improvement was the greatest among medical assistants, with a mean difference of 33.78% (See Table 2). Score improvement of medical assistants was, in particular, higher than that of the physicians participating in the course, a result that was statistically significant (*p* = 0.048).

### Results From Follow-Up

**Figure 2:**
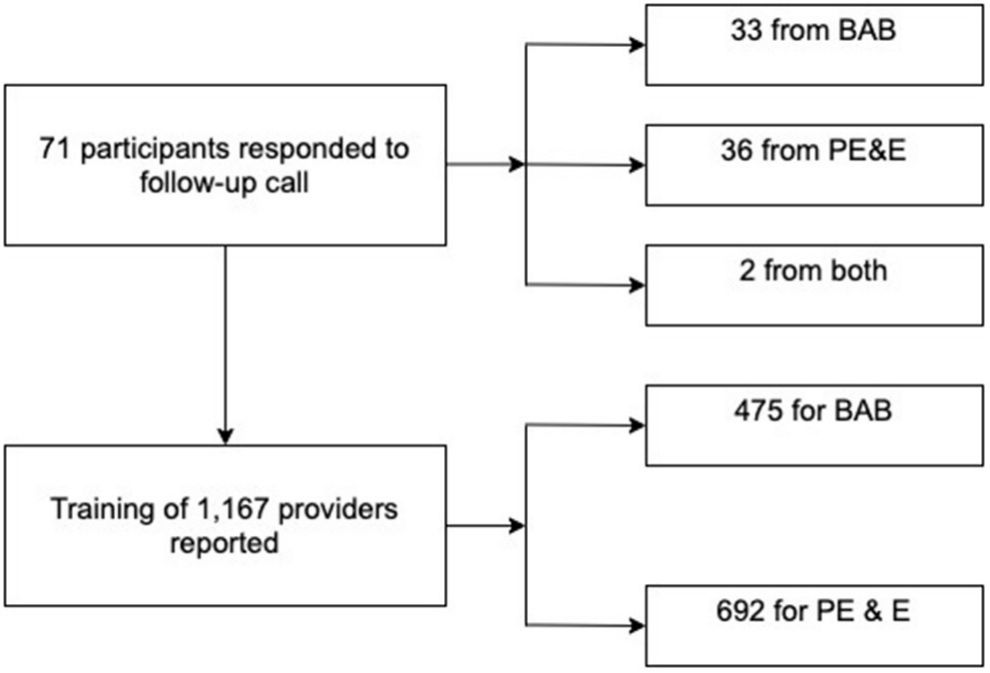
Flow chart of follow-up responses and subsequent training.

Seventy-one of the 132 master trainers responded to a phone call from the program staff to complete a follow-up questionnaire. Of these, 33 had participated in the BAB course, 36 in the PE&E course, and 2 in both courses. The questionnaire obtained information from each master trainer regarding the type and number of subsequent training workshops conducted, as well as the total number of participants trained. From the 71 respondents, a self-reported total of 1,167 healthcare providers were trained at subsequent workshops (Figure 2). Four hundred and seventy-five providers (40.7%) were trained in the BAB course, while 692 providers (59.3%) were trained in the PE&E course.

## Discussion

In the DR, broad access to skilled birth attendants paired with an unexpectedly high maternal mortality ratio suggests a need for capacity building of local healthcare workers to support best practices during and immediately following childbirth. Implementation of the HMS program represents a collaborative effort to empower local health care professionals to support peer knowledge acquisition and retention, reinforce clinical skills, and augment the availability of clinical and educational maternal resuscitation supplies. The HMS BAB and PE&E training curricula were deemed appropriate interventions for improving healthcare worker knowledge and skill levels due to demonstrated success from the implementation of the simulation-based neonatal resuscitation program, HBB, in the same region. The previously established partnerships and best practices from the HBB program allowed for a refined and efficient implementation process for the HMS program. Support from the Ministry of Health was founded on long-standing partnerships with the HBB leadership team, and recruitment of participating hospitals and providers was facilitated by previously established pilot sites for the HBB program rollout in this region. Invaluable support from Latter-day Saint Charities allowed for provision of training materials, clinical supplies, and logistical costs, while Latter-day Saint Charities and Jhpiego collaborated to complete the translation of PE&E course materials in time for program rollout. HMS regional program rollout was assisted by initial training workshops that were piloted in Santiago in December 2016. Challenges to widespread implementation with respect to scheduling, staffing, and space logistics were identified and addressed, allowing for the development of a strong foundation for HMS program expansion.

### Knowledge Tests and Score Improvement

Simulation-based training improved healthcare workers' knowledge of maternal care practices with respect to the diagnosis and management of both HMS topics, PPH and pre-eclampsia/eclampsia. A considerable improvement in knowledge was observed for both the BAB and PE&E workshops, with a 21.24% change and 30.25% change, respectively (Table 3). The larger percentage change observed for the PE&E curriculum correlates with a lower baseline pre-test score (44.28% compared to 68.26% for BAB), suggesting that the participants were initially less familiar with the topics covered in the PE&E curriculum. These results indicate that further reinforcement of the PE&E curriculum may be necessary, and comprehensive follow-up training should be conducted in order to ensure that knowledge acquisition is maintained and improved among participants over time. Overall, results from both curricula suggest that simulation-based training workshops have the potential to improve provider competency and eventually improve clinical practice in maternal care.

**Table 3:** Knowledge score improvement per HMS course observed as % change in score.

**Average score (%)**	**Both courses (%)**	**BAB (%)**	**PE&E (%)**
Pre-test score (%)	51.66	68.26	44.28
Post-test score (%)	79.3	89.5	74.53
% change	27.64	21.24	30.25

Although a notable improvement from pre-test to post-test was observed, a limitation in understanding knowledge acquisition and deficits among participants was the lack of question-level data available for analysis. Only raw scores of each participant's pre- and post-test were recorded rather than individual answer choices, and therefore analysis could not be performed regarding particular questions. Questions listed on the standardized knowledge tests may have been problematic due to confusing wording or inconsistent language compared to language used during the workshop. This lack of clarity could have led participants to answer questions incorrectly, even if knowledge of the topic was in fact present. This is further supported anecdotally, as multiple participants from training consistently mentioned specific questions that were difficult to understand. If the responses to each question were available, test questions yielding a high percentage of incorrect responses could have been removed, which would have provided a more clear understanding of true knowledge acquisition. Separately, the knowledge test for the BAB curriculum was previously only available from Jhpiego in English and required translation. These knowledge tests were translated into Spanish by a native Spanish-speaker and medical interpreter, however it is possible that the meaning of a question could have been skewed during the translation process or that there was discrepancy between the wording utilized during implementation of the workshops and wording utilized in the written test. Although it is unlikely that problems with translation contributed to lower knowledge scores due to utilization of native-language professionals for translation and subsequent review and piloting of the Spanish-language course materials by Dominican health care professionals, there is no evidence to support that it was not a contributing factor. Future training workshops should ensure that question-level data is recorded to facilitate more precise analysis and accurate conclusions regarding participant knowledge acquisition.

### Trend in Pre-test Knowledge Scores

**Figure 3:**
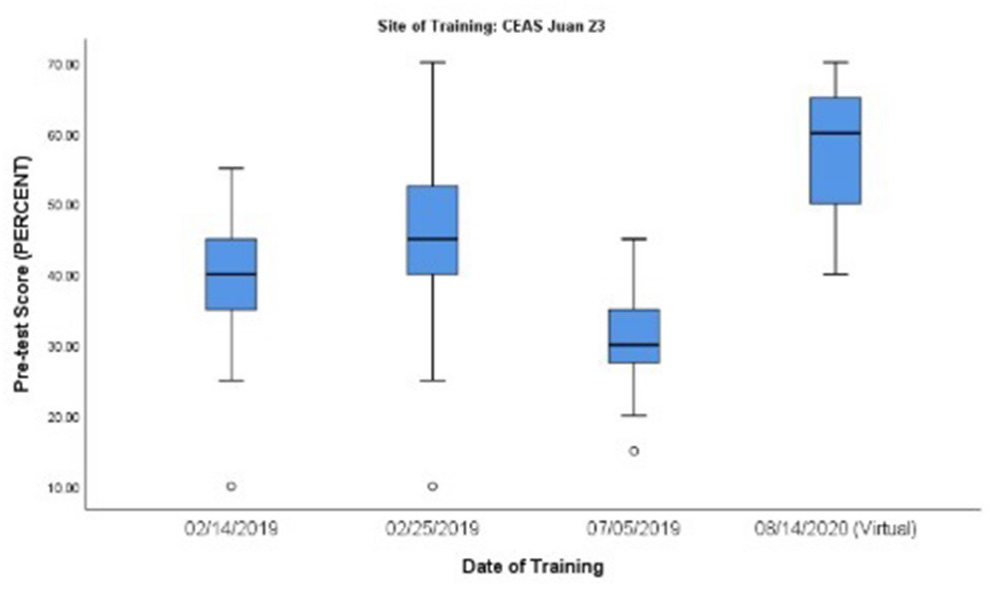
Mean PE&E pre-test scores over time at the CEAS Juan XXIII Hospital site.

Subanalysis of pre-test knowledge scores was conducted to investigate overall trends in general baseline (pre-test) knowledge over time in communities with HMS-trained professionals. The subanalysis compared mean pre-test scores over time at the following three sites: Santiago Master Training site, Barahona Master Training site, and CEAS Juan XXIII Hospital site. At the CEAS Juan XXIII Hospital site, an overall trend in improvement in PE&E pre-test scores over time among family medicine residents was observed, a result that was statistically significant (*p* < 0.001). This finding raises the question of whether *in situ* clinical practice and mentoring of master trainers and an increasing number of HMS-trained providers in a particular clinical site might contribute to increased baseline knowledge among their colleagues with respect to BAB and PE&E topics. While these preliminary results are encouraging, when analyzing all professional categories of course participants at the CEAS Juan XXIII Hospital site, the trend in improved baseline knowledge was no longer statistically significant and there was a notable outlier observed in a PE&E course taught in July 2019 (See Figure 3). Upon further investigation of participant data, the majority of participants from the outlier workshop identified as nurses (*n* = 6), while the remaining participants identified as internal medicine physicians (*n* = 5). This supports that the overall trend in improved baseline knowledge is specific to those specialized in family medicine at the CEAS Juan XXIII Hospital site, and that this group consistently performs better overall than other professions on HMS knowledge assessments. Although the overall results do follow an upward trend in increasing baseline PE&E knowledge, the subanalysis results do not draw statistical significance and merit further investigation. Proper investigation into overall improvement of baseline knowledge would provide valuable insight into the impact of the HMS program on the healthcare community throughout the DR.

While there is not currently enough data from any single training site to draw conclusions in terms of generalized baseline knowledge among communities with HMS-trained professionals over time, the subanalysis did confirm that regardless of site and profession, pre-eclampsia/eclampsia was a consistently less familiar topic for participants compared to PPH. Anecdotally, participants additionally shared that they felt more comfortable managing PPH in patients compared to pre-eclampsia/eclampsia, which provides further evidence of this trend and the need for reinforcement of the PE&E curriculum content after initial training.

### Skills Assessments

During training workshops, Objective Structured Clinical Examination (OSCE) checklists were used to perform formal skills assessments of the course participants. Although these checklists were used to guide and standardize the skills performed, an iterative training and evaluation approach was utilized to assess and reinforce skill acquisition, ensuring that participants were able to optimally perform each step of management algorithms and confidently perform the simulated skills correctly before completing the course. This method was used in place of collecting and recording individual skills assessment scores, as the use of scores is more susceptible to inter-rater reliability of the various trainers evaluating the participant and less coherent with the didactic approach described above.

Skills taught through the HMS BAB and PE&E curricula primarily emphasized medical knowledge rather than action-based skills. These skill sets involved following steps in standard protocols and identifying risk factors related to pre-eclampsia/eclampsia and PPH. Due to the nature of the skill sets and the use of an iterative training approach, no single skill set required additional emphasis or repetition. Anecdotally, participants did confirm that visual quantification of blood loss was challenging, which has been commonly observed and recorded in literature.

### Barriers to Data Collection

Data collection could have been improved in terms of categorization of participant professions. Thirteen professions were included; however, the largest group was physicians who did not specify their specialties (*n* = 95). This is largely due to the fact that early data collection forms used at initial master training sites (in 2016) utilized a list of categories that did not ask for physician specialty, while later forms used for participant training workshops were more detailed. Future workshops should utilize a standardized method of categorizing professions in order to obtain more accurate stratified results.

There were several challenges to collecting follow-up data. In general, contacting healthcare providers by phone is difficult due to demanding clinical schedules. Many respondents did not answer the phone even after multiple attempts. Additionally, the only staff member responsible for conducting follow-up interviews was the local program lead, which was a limiting factor in completing all of the follow-up calls at a single point in time. Finally, the onset of the COVID-19 pandemic interrupted the collection of follow-up data. The local program lead and master trainers became preoccupied with responding to the pandemic, and conducting follow-up interviews was no longer prioritized. At the time of writing this manuscript, follow-up interviews have not yet resumed.

### COVID-19 Pandemic Response

Beginning in March 2020, confirmed cases and deaths due to COVID-19 continued to climb throughout the Americas and the Caribbean (20). With much of the world in a state of lockdown, flexibility and innovation from the local team in the DR allowed for the continuation of the HMS training program despite pandemic restrictions. In August 2020, a virtual training workshop for the PE&E curriculum was piloted with participants from CEAS Juan XXIII Hospital in Santiago, DR. This workshop was conducted via the platform Google Hangouts, and did not include the practical element of the simulation training due to limitations of the virtual medium and requirements in physical distancing. The knowledge tests were administered via Google Forms, and participants were expected to complete the post-test within a few days of attending the workshop. The analysis showed no significant difference in knowledge acquisition between in-person and virtual training workshops. These results suggest that virtual training workshops have the potential impact knowledge acquisition as effectively as the in-person workshops. These promising results are limited by the small sample size in virtual training and merit further investigation as more data is collected from ongoing virtual training. While these results are promising, the increased baseline scores observed from the virtual training session may have limited opportunities for improvement and overestimated the effectiveness of the training. It is also important to note that the increased average pre-test scores observed for this workshop could have been due to the fact that this hospital is considered a model training site, as it is the home site of the local program lead and has hosted the greatest number of training workshops out of all 13 sites. Additionally, the cohort from this workshop consisted of family medicine residents, which aligns with the trend that family medicine physicians perform better on the knowledge assessments compared to other professions observed in this study.

Concerns surrounding the limitations of conducting virtual workshops were considered. First, as this virtual workshop did not include a hands-on practical skills component, it is possible that the virtual workshop will not effectively impact skill acquisition and clinical practice, aligning with the idea that without opportunities for hands-on practice and simulation, clinical practice will not change. Additionally, a potential issue for participants is the requirement of access to a stable internet connection and technology, potentially limiting the reach and overall impact of the program. For those with proper access to these resources, the internet may still be unreliable, which could cause participants to miss portions of the training, and therefore score lower on knowledge assessments. These are valid concerns that require further investigation, however in light of the ongoing COVID-19 pandemic, these preliminary results provide evidence that conducting virtual workshops may be a viable alternative to in-person training at this time.

### Next Steps

The continuation of the COVID-19 pandemic will largely impact the next steps of this regional training program. Once it is safe to resume in-person program activities, other aspects of capacity building should be investigated such as skill attainment during the simulated portion of the workshops, as well as perceived confidence levels before and after training. These characteristics were not investigated in this evaluation, however immediate and long-term results of how skill and confidence levels are impacted by the training are crucial to understanding the true effectiveness of the HMS program. Additionally, once it is possible, more extensive follow-up interviews with participants should be conducted to assess its long-term influence on knowledge levels. Continued follow-up training with those trained as master trainers should also be conducted in order to assess the sustainability of the program and the success of the T3 methodology. Further information on the number of training sessions held by master trainers in their respective areas could identify potential gaps or barriers to the continuation of training that can be addressed through future programmatic updates. Finally, to improve evaluation of the impact of the training curricula, per-question knowledge test data will be collected and analyzed in order to better gauge provider understanding of the workshop content and identify problematic components of data collection tools. With these proposed changes, evaluation of the HMS program in the DR will be able to better and more comprehensively assess the impact of training on healthcare providers and confirm the validity of using simulation-based training to address maternal mortality through knowledge and skill acquisition.

## Conclusion

Simulation-based training is an important maternal health intervention that can be feasibly and sustainably implemented in low-resource settings. Findings from implementation of the HMS BAB and PE&E training curricula in the DR show a significant improvement in knowledge acquisition of participant healthcare workers. There is also preliminary evidence that virtual training workshops may be as effective in improving participants' knowledge acquisition as in-person training workshops, which is particularly relevant for the implementation of this and similar programs during the ongoing COVID-19 pandemic. Expanded skill reinforcement in addition to improved knowledge levels have the potential to positively impact local clinical practice. This evaluation demonstrates the importance of strong, collaborative partnerships for the sustainability of health training programs. Further, it is evident that leadership from local program leaders is vital to the development and sustainability of the described programmatic experience in the DR. These promising findings and insights encourage the feasible scale-up of maternal health research and interventions in the DR and in other low-resource settings throughout Latin America and the Caribbean.

## Data Availability Statement

The raw data supporting the conclusions of this article will be made available by the authors, without undue reservation.

## Author Contributions

MS wrote the first draft. AL, WR, EJ, DL, JF, CC, SB, and LJ contributed revisions and were intrinsic in the program design, development, and implementation. MS conducted the program evaluation, while TV conducted data analysis for the evaluation. All authors reviewed the final draft and approved the final manuscript.

## Conflict of Interest

The authors declare that the research was conducted in the absence of any commercial or financial relationships that could be construed as a potential conflict of interest.
