# Supplementary material for: Helping Mothers Survive: Program Evaluation and Early Outcomes of Maternal Care Training in the Dominican Republic
Source: Front Public Health. 2021 Jun 16;9:660908. doi: 10.3389/fpubh.2021.660908 (PMC8242252; doi:10.3389/fpubh.2021.660908)
Supplement: Supplementary file 1 [file Data_Sheet_1.PDF]

Pre-test / Post-test (circle one)

Helping Mothers Survive: Bleeding after Birth Complete Day 1 Knowledge Assessment

Participant name or ID# ______________________ Date ________________

INSTRUCTIONS: For each question, circle the letter for one correct answer.

1. Which of the following lists contain the three MAIN causes of bleeding after birth?

   a. Soft uterus, a retained placenta, and perineal tearing
   b. Soft uterus, malaria, and dehydration
   c. Retained placenta, malaria, and perineal tearing
   d. Soft uterus, twins, and perineal tearing

2. Which of the following lists contain the three parts of Active Management of the Third Stage of Labor?

   a. Give uterotonic, manually remove the placenta, and check for tears
   b. Cut the cord, wait for the placenta to deliver, and give uterotonic
   c. Give uterotonic, provide controlled cord traction, and check tone of the uterus
   d. Wait for the placenta to deliver, check for tears, and check the tone of the uterus

3. According to the World Health Organization, a uterotonic medication should be given to the mother:

   a. Immediately before delivery of the baby
   b. Within 1 minute of delivery of the baby
   c. Within 5 minutes of delivery of the baby
   d. Within 1 hour of delivery of the baby

4. Which medication does not have any temperature requirements for storage?

   a. Misoprostol
   b. Oxytocin
   c. Ergometrine

5. Which of the following uterotonic medication doses is CORRECT for Active Management of Third Stage of Labor?

   a. Misoprostol—800 mcg
   b. Misoprostol—1200mcg
   c. Oxytocin—10 units
   d. Oxytocin—40 units

6. How often should a woman's bleeding and uterine tone be checked after delivery of the placenta?

   a. Every 10 minutes for the first 6 hours after delivery
   b. Every 15 minutes for the first 2 hours after delivery
   c. Once an hour for the first 24 hours
   d. Once a day for the first week after delivery

7. If a mother is bleeding, but her uterus is hard and her placenta is out and complete, which of the following actions is MOST correct?

   a. Check for tears
   b. Give a second dose of uterotonic
   c. Fill the mother's vagina with gauze
   d. Ask the mother if she has HIV

8. If you have not been trained to suture, and if the mother is bleeding from tears that you can see, which of the following actions is MOST correct?

    a. Fill the mother's vagina with clean gauze
    b. Use clean technique to apply steady pressure with clean gauze
    c. Massage her uterus to stop the bleeding

9. If the mother's placenta is out and intact, her uterus is soft and does not respond to massage, she has received 2 doses of uterotonic and you can't see any tears, and she continues to bleed excessively the MOST correct thing to do next is:

    a. Have her squat and bear down
    b. Give her antibiotics
    c. Offer her something to eat
    d. Provide bi-manual compression

10. When transporting a patient to a higher level of care facility you should leave the baby with the family.

    a. True
    b. False

11. If the uterus is hard, the mother cannot have a postpartum hemorrhage.

    a. True
    b. False

12. What kind of postpartum care is MOST CORRECT for a mother who has received bimanual compression for excessive bleeding?

    a. The same kind of care as any other mother. If the bleeding has stopped she is fine.
    b. Advanced care because she has lost a lot of blood and may bleed again
    c. Home care as soon as possible after delivery so that she can rest
    d. Advanced care so that her uterus can be removed

13. If the mother's placenta is out and intact, her uterus is hard, and you can't see any tears, but she continues to bleed excessively, you must get an advanced care provider to assist with caring for the mother.

    a. True
    b. False

14. A slow flow of blood from the vagina after delivery that does not stop can be a sign of a postpartum hemorrhage.

    a. True
    b. False

15. If the placenta has not delivered within 1 hour or the mother is bleeding too much, advanced care should be obtained immediately.

    a. True
    b. False
