# Supplementary material for: Helping Mothers Survive: Program Evaluation and Early Outcomes of Maternal Care Training in the Dominican Republic
Source: Front Public Health. 2021 Jun 16;9:660908. doi: 10.3389/fpubh.2021.660908 (PMC8242252; doi:10.3389/fpubh.2021.660908)
Supplement: Supplementary file 2 [file Data_Sheet_2.PDF]

Helping Mothers Survive: Knowledge Test PE&E

Participant ID# ____________________ Date ____________________

Is this a pre-training or post-training assessment? (Circle one.) **a. PRE** **b. POST**

INSTRUCTIONS: For each question, circle the letter for the correct answer.

Day 1 Items

1. What level of proteinuria on a urine dipstick would make you suspicious that a woman has pre-eclampsia? Select all correct answers.

    a. 1+ reading on urine dipstick
    b. 2+ reading on urine dipstick
    c. 3+ reading on urine dipstick

2. When should a provider check the blood pressure of a pregnant or post-partum woman?

    a. During all visits and labor
    b. If the woman complains of blurred vision or headache
    c. During all visits and labor OR if the woman complains of blurred vision or headache

3. A woman presenting at 26 weeks' gestation with a blood pressure of 164/98 and proteinuria of 2+ would be suspected of?

    a. Pre-eclampsia
    b. Severe pre-eclampsia
    c. Eclampsia

4. A woman presents for the first time to your clinic at 30 weeks with a blood pressure of 144/92 and 3+ proteinuria. When should you retake her blood pressure to see if she has pre-eclampsia.

    a. 1 hour
    b. 4 hours
    c. 12 hours

5. Which scenario below indicates the diagnosis of severe pre-eclampsia in a woman at 32 weeks gestation?

   a. BP of 152/96, proteinuria 2+, headache relieved with paracetamol
   b. BP 144/94, severe headache, visual changes, convulsions, and oliguria
   c. BP 152/90, severe headache, right upper quadrant pain and visual changes

6. What is the first thing you will do if a pregnant woman is having an eclamptic convulsion?

   a. Check blood pressure
   b. Shout for help to mobilize the team
   c. Give loading dose of magnesium sulfate

7. How often would you monitor a woman with pre-eclampsia at 35 weeks gestational age who lives nearby your clinic, if her laboratory tests are normal and her fetus is in good condition?

   a. Weekly
   b. Continuously in a hospital setting
   c. Twice a week

8. What uterotonic medication should never be given to a woman diagnosed with hypertension?

   a. Ergometrine
   b. Oxytocin
   c. Misoprostol

9. The World Health Organization's recommended IV/IM regimen of magnesium sulfate (MgSO4) loading dose is comprised of:

   a. 5 g MgSO4 20% solution IV plus 5 g MgSO4 50% solution with lignocaine IM in each buttock
   b. 4 g MgSO4 50% solution IV plus 5 g MgSO4 20% solution with lignocaine IM in each buttock
   c. 4 g MgSO4 20% solution IV plus 5 g MgSO4 50% solution with lignocaine IM in each buttock

10. The goal of antihypertensive medication is to maintain diastolic blood pressure within what range?

    a. Between 100 and 110 mmHg
    b. Between 80 and 100 mmHg
    c. Between 90 and 100 mmHg

11. Treatment for severe pre-eclampsia may save the life of an unborn child, therefore pregnant women do not have the right to refuse treatment.

   a. True
   b. False

12. The first day of Mrs. C's last menstrual period was 22 Sept. Today is 20 April. What is her gestational age today?

   a. 29 weeks, 4 days
   b. 30 weeks, 1 day
   c. 31 weeks, 1 day

Additional Questions for Helping Mothers Survive PE/E Day 2

13. If available, which laboratory tests should be done on women with suspected pre-eclampsia?

   a. CBC with platelet count, liver enzymes, and serum creatinine
   b. CBC with platelet count, liver enzymes, and hemoglobin
   c. CBC with platelet count, serum creatinine, and uric acid

14. How often should you monitor a woman receiving MgSO4?

   a. Every 15 minutes
   b. Every 30 minutes
   c. Every hour

15. What reason would make you withhold the next scheduled dose of MgSO4?

   a. Patellar reflexes are absent
   b. Respiratory rate is 16 breaths per minute
   c. Blood pressure is 138/88

16. What should you confirm before deciding to provide expectant management and delay childbirth for a woman with severe pre-eclampsia at 33 weeks' gestation?

   a. Hypertension is well controlled, there are no signs of worsening maternal status, the fetus is tolerating pregnancy, and the woman and fetus can be closely monitored
   b. Hypertension is well controlled, there are no signs of worsening maternal status, the fetus is tolerating pregnancy, but the woman and fetus cannot be closely monitored
   c. Hypertension is well controlled, there are no signs of worsening maternal status, there are signs of fetal distress, but the woman and fetus can be closely monitored.

17. What are the gestational ages between which you would give antenatal corticosteroids (such as dexamethasone) to improve neonatal outcomes?

    a. Between 28 and 36 weeks
    b. Between 24 and 34 weeks
    c. Between 34 and 36 weeks

18. Women with eclampsia should be delivered within how many hours of diagnosis?

    a. 8 hours
    b. 12 hours
    c. 24 hours

19. How long should MgSO4 therapy continue after birth?

    a. For 12 hours after birth or after the last convulsion, whichever occurs last
    b. For 24 hours after birth or after the last convulsion, whichever occurs last
    c. For 24 hours after birth or after the last convulsion, whichever occurs first

20. Your patient received a loading dose of magnesium sulfate 30 minutes ago. You hear shouting and rush over to see that she is having a convulsion. What medication are you going to give her for this?

    a. Antihypertensive
    b. Magnesium sulfate 50% solution 4g (1g/2ml x 2) by IV over 5 minutes
    c. Magnesium sulfate 20% solution 2g (1g/2ml x 2) by IV over 5 minutes
